# Supplementary material for: COZOID: contact zone identifier for visual analysis of protein-protein interactions
Source: BMC Bioinformatics. 2018 Apr 6;19:125. doi: 10.1186/s12859-018-2113-6 (PMC5889581; doi:10.1186/s12859-018-2113-6)
Supplement: Supplementary file 2 — Software build. Executable binary file of the software tool. (ZIP 118,784 kb) [file 12859_2018_2113_MOESM2_ESM.zip › cozoid/platform/docs/testng-6.5.1-javadoc.zip!/org/testng/xml/XmlInclude.html]

XmlInclude (TestNG)


---


|  |  |  |
| --- | --- | --- |
| **Overview** \| **Package** \| **Class** \| **Tree** \| **Deprecated** \| **Index** \| **Help** | |  |
| **PREV CLASS**   **NEXT CLASS** | **FRAMES**    **NO FRAMES**     **All Classes** |  |
| SUMMARY: NESTED \| FIELD \| CONSTR \| METHOD | DETAIL: FIELD \| CONSTR \| METHOD |  |


---


## org.testng.xml Class XmlInclude

```
java.lang.Object
  org.testng.xml.XmlInclude
```

**All Implemented Interfaces:**: java.io.Serializable

---

``` public class XmlInclude extends java.lang.Object implements java.io.Serializable ```

**See Also:**: Serialized Form

---

**Constructor Summary**

`XmlInclude(java.lang.String n)`

`XmlInclude(java.lang.String n,
int index)`

`XmlInclude(java.lang.String n,
java.util.List<java.lang.Integer> list,
int index)`


| **Method Summary** | |
| --- | --- |
| `boolean` | `equals(java.lang.Object obj)` |
| `java.lang.String` | `getDescription()` |
| `int` | `getIndex()` |
| `java.util.List<java.lang.Integer>` | `getInvocationNumbers()` |
| `java.lang.String` | `getName()` |
| `java.util.Map<java.lang.String,java.lang.String>` | `getParameters()` |
| `int` | `hashCode()` |
| `void` | `setDescription(java.lang.String description)` |
| `void` | `setParameters(java.util.Map<java.lang.String,java.lang.String> parameters)` |
| `java.lang.String` | `toXml(java.lang.String indent)` |

**Methods inherited from class java.lang.Object**

`clone, finalize, getClass, notify, notifyAll, toString, wait, wait, wait`

**Constructor Detail**

### XmlInclude

```
public XmlInclude(java.lang.String n)
```

---


### XmlInclude

```
public XmlInclude(java.lang.String n,
                  int index)
```

---


### XmlInclude

```
public XmlInclude(java.lang.String n,
                  java.util.List<java.lang.Integer> list,
                  int index)
```


**Method Detail**

### setDescription

```
public void setDescription(java.lang.String description)
```

---


### getDescription

```
public java.lang.String getDescription()
```

---


### getName

```
public java.lang.String getName()
```

---


### getInvocationNumbers

```
public java.util.List<java.lang.Integer> getInvocationNumbers()
```

---


### getIndex

```
public int getIndex()
```

---


### toXml

```
public java.lang.String toXml(java.lang.String indent)
```

---


### hashCode

```
public int hashCode()
```

:   **Overrides:**: `hashCode` in class `java.lang.Object`

---


### equals

```
public boolean equals(java.lang.Object obj)
```

:   **Overrides:**: `equals` in class `java.lang.Object`

---


### setParameters

```
public void setParameters(java.util.Map<java.lang.String,java.lang.String> parameters)
```

---


### getParameters

```
public java.util.Map<java.lang.String,java.lang.String> getParameters()
```


---


|  |  |  |
| --- | --- | --- |
| **Overview** \| **Package** \| **Class** \| **Tree** \| **Deprecated** \| **Index** \| **Help** | |  |
| **PREV CLASS**   **NEXT CLASS** | **FRAMES**    **NO FRAMES**     **All Classes** |  |
| SUMMARY: NESTED \| FIELD \| CONSTR \| METHOD | DETAIL: FIELD \| CONSTR \| METHOD |  |


---
